# Sex differences in dietary consumption and its association with frailty among middle-aged and older Australians: a 10-year longitudinal survey

**DOI:** 10.1186/s12877-021-02165-2

**Published:** 2021-03-31

**Authors:** Xiaoyue Xu, Sally C Inglis, Deborah Parker

**Affiliations:** 1 School of Population Health, University of New South Wales, Sydney, New South Wales Australia; 2 The George Institute for Global Health, Sydney, New South Wales Australia; 3 Improving Palliative, Aged and Chronic Care through Clinical Research and Translation Research Centre, Faculty of Health, University of Technology Sydney, Sydney, New South Wales Australia

## Abstract

**Background:**

Nutritional status has been considered as a key factor in preventing the development of the frailty syndrome. However, sex-specific dietary consumption transition over time and how it impacts of frailty status are unclear.

**Method:**

We assessed 113,039 adults (aged 50 years and over) from the 45 and Up Study who had completed both baseline (2006–2009) and follow-up (2012–2015) surveys. Dietary consumption was assessed by a short food frequency questionnaire. Frailty was identified by the FRAIL scale. Multinomial regression models were used to examine the association between a long-term dietary consumption and frailty, stratified by sex.

**Results:**

Of a total of 113,039 participants, females had a higher percentage of pre-frailty and frailty than males (pre-frailty: 35.5% for female and 30.1% for male; frailty: 4.86% for female and 3.56% for male). As age increased, males had significant decreases in overall dietary risk scores, while females had significant increases in overall dietary risk scores. Males and females with a long-term consumption of adequate fruits, high grains or had a variety of foods were related to a low risk of frailty. Females with a long-term consumption of adequate vegetables or high lean meats and poultry were related to a low risk of frailty. Females with an unhealthy diet at both surveys [Relative Risk Ratio (RRR) = 1.32, 95% CI: 1.18; 1.49], and those with unhealthy diet at either surveys (RRR = 1.28, 95% CI: 1.12; 1.47, RRR = 1.19, 95% CI: 1.04; 1.37) had a higher risk of frailty compared to those had a long-term healthy diet. No association were found between overall dietary risk and frailty for males.

**Conclusion:**

Males and females changed their dietary consumption as they age. These changes affect its association with frailty, particularly for females. Sex-specific dietary advice in prevention of frailty needs to be further developed.

**Supplementary Information:**

The online version contains supplementary material available at 10.1186/s12877-021-02165-2.

## Background

The prevalence of frailty is higher in older adults but is not considered as a part of normal ageing. Frailty is a medical condition characterised by a functional (physical and cognitive) decline, that requires the need for assistance to perform the daily living activities [1]. There is no curative treatment for frailty, so the efforts have focused on the prevention and palliation of symptoms, with focus on effective physical and nutritional interventions or control of polypharmacy [2].

The factors responsible for the development of frailty is still a matter of intense debate, but there have been several factors noted, such as sarcopenia or muscle mass loss. In this line, nutritional status has been identified as a key factor in preventing the development of frailty syndrome. Previous studies predominantly cross-sectional in design, have provided evidence on the relationship of micronutrients (e.g. vitamins D, E and C, and folate), macronutrient (e.g. protein), dietary pattern (e.g. Mediterranean diet), dietary quality and frailty [3]. Overall, a diet with low energy intake, insufficient consumption of protein, micronutrients such as vitamin D, C, calcium and omega-3 fatty acids is associated with an increased risk of frailty development [4]. Control or balance in nutritional status is essential to prevent sarcopenia and further frailty development [3, 4]. The relationship between nutrition and frailty is likely bidirectional that poor nutrition or malnutrition might contribute to frailty, or conversely, frailty may contribute to poor nutrition or malnutrition [5–7].

As people age, they may eat less and make difference food choices. It is unclear whether the dietary consumption transition over the life course may impact on the frailty status of older individuals. In addition, studies have indicated sex/gender differences in food choice, and in energy and nutrients intake [8]. It is also possible that dietary consumption may differ by sex as people age, which may have different impacts on frailty status. Although it has been clearly stated in the previous research that sex-specific analyses of research data should be the norm [9], most studies failed to report this, both in Australia and internationally.

To better understand the sex-specific frailty status among middle-aged and the older Australian population, also to understand the dietary consumption transition and how it impacts of frailty status, this study used data from the longitudinal 45 and Up Study to 1) evaluate sex-specific frailty status, 2) track changes of dietary consumption by frailty status and sex, and 3) examine the association between long-term dietary consumption and frailty, stratified by sex.

## Method

### Study population

The Sax Institute 45 and Up Study baseline and follow-up data were used. The 45 and Up Study is the largest ongoing study of healthy ageing ever undertaken in the Southern Hemisphere and is designed to understand how Australians are ageing. Participants were randomly sampled from the Department of Human Services enrolment database [10]. A total of 267,153 men and women aged 45 and over across New South Wales, Australia were recruited and surveyed in 2006–2009, representing about 10% of this age group. Upon recruitment, participants provided consent for future follow-up. The first follow-up survey data were collected between 2012 and 2015. At both time points, socioeconomic, health behaviour and health related information were collected via a comprehensive questionnaire that was mailed to people. Details of the 45 and Up Study sampling process are described elsewhere [11]. The baseline and follow-up questionnaires are available at Sax Institute website (https://www.saxinstitute.org.au/our-work/45-up-study/questionnaires/). In the present study, we included those participants who completed both baseline and follow-up questionnaires on dietary consumption. A total of 113,039 participants were included in the analysis.

### Outcome variable: frailty

We used the FRAIL scale to identify frailty. The FRAIL scale was developed by the Geriatric Advisory Panel of the International Academy of Nutrition and Aging, and can be administered by conducting a brief interview or constructed from self-reported survey data [12]. Previous studies have shown that the FRAIL scale score is predictive of mortality and disability [12], and the scale has been tested as a valid and responsive tool to identify frailty with suitability for use in longitudinal studies of older Australians [13].

The FRAIL scale is based on deficits in five domains: Fatigue; Resistance (ability to climb one flight of stairs); Ambulation (ability to walk one block), Illnesses (greater than 5) and Loss of Weight (> 5%). Participants were scored positive as 1 for each of these five domains, so the FRAIL ranges from 0 (not frail) to 5 (most frail) [13, 14]. The FRAIL scale was used to categorize participants as healthy (score 0), pre-frail (score of 1–2) or frail (score of 3–5).

These five domains are matched with the 45 and Up study questionnaire. Specifically, for ‘Fatigue’: participants scored positive if they responded, “most of the time” or “all of the time” to the questionnaire “during the past 4 weeks, about how often did you feel tired out for no good reason?” For ‘Resistance’: participants scored positive if they responded “limited a little” or “limited a lot” on their ability to climb one flight of stairs. For ‘Ambulation’: participants scored positive if they responded “limited a little” or “limited a lot” on their ability to walk 100 m. For ‘Illness’: if participants reported more than five of the following diseases: Alzheimer’s diseases or dementia, angina pectoris or heart attack, depression, arthritis (including osteoarthritis and rheumatoid arthritis), asthma, bronchitis or emphysema, diabetes, hypertension, osteoporosis and stroke. For ‘Loss of weight’: participants scored positive if their self-reported weight decreased by 5% or more between baseline and follow-up survey. The domains of ‘Fatigue’, ‘Resistance’, ‘Ambulation’ and ‘Illnesses’ were identified from follow-up data. ‘Loss of weight’ was identified from both baseline and follow-up data. The frailty status was identified at follow-up data based on all of five domains.

### Predictive variable: a long-term dietary consumption

Dietary consumption was identified from seven food components based on the Australian Dietary Guideline (ADG): vegetable, fruit, grains, lean meat and poultry, dairy, food diversity, and alcohol consumption [15, 16]. In the 45 and Up questionnaire, dietary consumption was assessed by short food frequency questions, which have been described in previous research [17, 18]. Each of the questions on diet were previously validated in the Million Women Study [19].

Adequate fruit and vegetable (FV) consumption was identified according to the ADG. Adequate vegetable consumption was identified as ≥5.5 serves per day for males aged 51–70 years, ≥5 serves per day for males who aged 70 and above; and ≥ 5 serves per day for females across all age groups. Adequate fruit consumption was identified as ≥2 serves per day for males and females across all age groups. The frequency of food groups for grains, and lean meat and poultry were divided into two groups: lower than mean (0–5 times per week for grains, 0–7 times per week for lean meats and poultry) and higher than mean (> 5 times per week for grains, ≥7 times per week for lean meats and poultry). Dairy was categorised as Yes/No. Food diversity was identified if participants consumed all five food groups, i.e., fruit, vegetable, grains, lean meat and poultry, and dairy. Alcohol consumption was identified as Yes/No. Based on ADG, we further generated overall dietary risk scores (Table S1), with range from 0 (the healthiest dietary consumption) to 9 (the unhealthiest dietary consumption).

To evaluate a long-term dietary consumption for each food group and overall dietary consumption, we used both baseline and follow-up dietary data and grouped the participants into four sub-groups (Table S2). In brief, four sub-groups were: a. had healthy diet at baseline and follow-up (+, +), b. had unhealthy diet at baseline and follow-up (−,-), c. had healthy diet at baseline but unhealthy diet at follow-up (+,-), and d. had unhealthy diet at baseline but healthy diet at follow-up (−,+). The detailed explanation for sub-group classifications for each food group and overall dietary risk are shown in Table S2.

### Covariates

We included socio-demographic factors and health behaviour factors as covariates in the analysis. Socio-demographic included age, country of birth, marital status, education and socioeconomic level, and health behaviour factors included smoking and physical activity levels. Country of birth was categorized as Australian versus other countries. Education levels were divided into three categories, i.e., Low: no school certificate or other qualification, and school or intermediate certificate; Medium: high school or leaving certificate; and trade or apprenticeship; and High: certificate or diploma, and university degree or higher. Socioeconomic levels were assessed by Socio-Economic Indexes for Areas (SEIFA), which is based on three quantiles (low, medium, high) of Index of Relative Socio-economic Advantage and Disadvantage [20].

Smoking was identified as never smoke, previous smoker, and current smoker, based on two questions of “Have you ever been a regular smoker?” and “Are you a regular smoker now?”. Physical activity was measured using the Active Australia Survey, asking the total time spent on walking, moderate-intensity, and vigorous-intensity physical activity in the previous week. Adequate physical activity was identified if people spent 150 min of moderate intensity physical activity, or 75 min of vigorous intensity physical activity per week [21].

### Statistical analysis

N (%) were used to present the participants’ characteristics by three FRAIL categories for males and females. Chi-square tests were used to examine statistical differences between three FRAIL categories and socio-demographic factors for males and females, respectively. The statistical differences between males and females for the prevalence of five FRAIL domains were tested by Chi-square. T-test was used to test mean differences of food group consumption (continuous variables), and Chi-square was used to test differences for categorical food group consumption across baseline and follow-up for males and females, respectively. Multinomial regression models were used to test 1) the association between a long-term dietary consumption of each food group and frailty, stratified by sex; and 2) the association between a long-term overall dietary consumption and frailty, stratified by sex. These results are reported in the tables, with Relative Risk Ratios (RRR) and 95% Confidence Interval (CI) in two models: 1) crude model and 2) adjusted model that after adjustment of socioeconomic factors and health behaviour factors. All analyses were conducted in STATA/SE 14 (StataCorp, USA).

## Results

### Participants characteristics

**Fig. 1:**
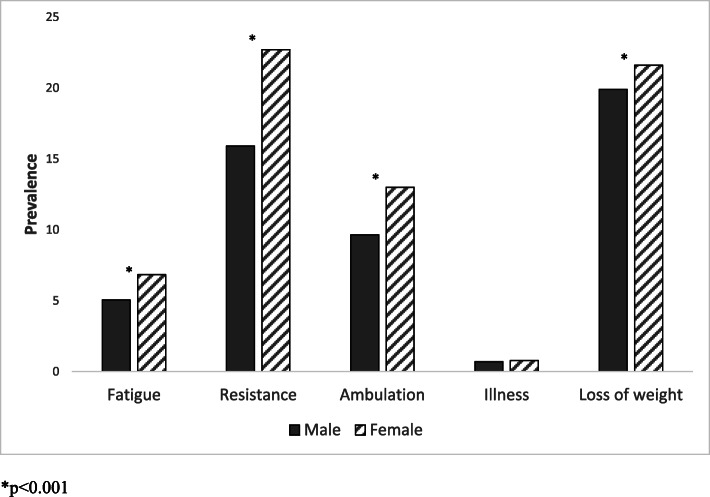
Prevalence of five domains of the FRAIL scale by sex. *p < 0.001

There were 15,745 (30.1%) males and 21,543 (35.5%) females identified as pre-frail, 1864 (3.56%) males and 2948 (4.86%) females were identified as frail. Participants characteristics by three FRAIL categories for males and females were shown in Table 1. Overall, females had a higher percentage of pre-frailty and frailty than males across all socioeconomic factors. Females who aged 80 years and over, widowed, with low education level and lived in low socioeconomic areas had higher percentage of pre-frailty and frailty than male counterparts. Females, who were identifies as per-frail and frail, had higher percentage of CVD, diabetes, and higher overall dietary risks than males. Across five domains of the FRAIL scale, females had a higher percentage of fatigue, resistance, ambulation, and loss of weight than males (*p* < 0.001). No association were found between males and females on illness (*p* = 0.12) (Fig. 1).

**Table 1:** Participants characteristics by three FRAIL categories for males and females (*N* = 113,039)

	Males			***p*** value	Females			***p*** value
	Healthy	Pre-frail	Frail		Healthy	Pre-frail	Frail	
**Age group**		N (%)				N (%)		
50–64 years	12,482 (74.3)	4026 (24.0)	298 (1.77)	< 0.001	16,179 (68.2)	6917 (29.2)	628 (2.65)	< 0.001
65–79 years	18,514 (67.5)	8081 (29.5)	847 (3.09)		17,527 (59.3)	10,795 (36.5)	1245 (4.21)	
80 years +	3770 (46.4)	3638 (44.8)	719 (8.85)		2467 (33.5)	3827 (51.9)	1074 (14.6)	
**Country of birth**								
Australia	26,397 (66.3)	11,959 (30.0)	1452 (3.65)	0.07	28,349 (59.5)	16,924 (35.5)	2369 (4.97)	< 0.01
Other countries	8187 (66.9)	3656 (29.9)	394 (3.22)		7664 (60.5)	4445 (35.1)	553 (4.37)	
**Marital status**								
Married/partner	28,778 (68.3)	12,043 (28.6)	1286 (3.05)	< 0.001	25,719 (64.1)	13,014 (32.5)	1367 (3.41)	< 0.001
Single/divorce/separated	3951 (61.8)	2141 (33.5)	306 (4.78)		5898 (56.5)	3912 (37.5)	637 (6.10)	
Widowed	1604 (50.1)	1357 (42.3)	244 (7.61)		4271 (44.5)	4426 (46.1)	907 (9.44)	
**Education**								
Low	6830 (58.6)	4164 (35.7)	664 (5.70)	< 0.001	12,056 (53.2)	9049 (39.9)	1556 (6.87)	< 0.001
Medium	16,369 (66.3)	7404 (30.0)	905 (3.67)		13,602 (61.0)	7704 (34.6)	981 (4.40)	
High	11,205 (72.6)	3976 (25.8)	262 (1.70)		10,221 (67.6)	4537 (30.0)	352 (2.33)	
**SEIFA**^**a**^								
Low	10,119 (61.5)	5493 (33.4)	856 (5.20)	< 0.001	11,030 (54.3)	7870 (38.8)	1411 (6.95)	< 0.001
Medium	10,944 (66.7)	4896 (29.9)	559 (3.41)		11,383 (60.1)	6702 (35.4)	843 (4.45)	
High	11,693 (70.8)	4461 (27.0)	365 (2.21)		11,731 (65.0)	5783 (32.0)	545 (3.02)	
**Dietary risk scores**								
Lower than mean	11,369 (65.4)	5333 (30.7)	671 (3.86)	0.001	16,082 (60.1)	9476 (35.4)	1192 (4.46)	< 0.001
Higher than mean	23,397 (66.8)	10,412 (29.8)	1193 (3.41)		20,091 (59.2)	12,067 (35.6)	1756 (5.18)	
**Alcohol consumption**								
No	6849 (57.1)	4397 (36.7)	749 (6.24)	< 0.001	12,157 (50.9)	9908 (41.5)	1828 (7.7)	< 0.001
Yes	27,318 (69.4)	10,980 (27.9)	1056 (2.68)		23,215 (66.1)	10,927 (31.1)	971 (2.77)	
**Cardiovascular disease**								
No	25,435 (71.9)	9236 (26.1)	694 (1.96)	< 0.001	30,458 (64.7)	15,267 (32.4)	1382 (2.93)	< 0.001
Yes	9331 (54.9)	6509 (38.3)	1170 (6.88)		5715 (42.2)	6276 (46.3)	1566 (11.6)	
**Diabetes**								
No	30,806 (69.1)	12,533 (28.1)	1270 (2.85)	< 0.001	34,056 (61.8)	18,813 (34.1)	2250 (4.08)	< 0.001
Yes	3428 (48.9)	3003 (42.8)	582 (8.30)		1976 (37.2)	2653 (49.9)	689 (13.0)	

^a^*SEIFA* Socio-Economic Indexes for Areas

### The changes of dietary consumption by frailty status and sex

The changes of each food group consumption across two waves by three FRAIL categories for males and females were shown in Table 2. Overall, we found similar trends in food consumption across three FRAIL categories. This may be due to the overall dietary behaviour changes as peoples’ age.

**Table 2:** The changes of dietary consumption across baseline and follow-up by three FRAIL categories for males and females

	Males				Females			
		Baseline	Follow-up	***P*** value		Baseline	Follow-up	***P*** value
**Vegetable**	N	Mean (SD)	Mean (SD)		N	Mean (SD)	Mean (SD)	
Health	34,766	3.45 (2.58)	3.59 (2.85)	< 0.001	36,173	4.44 (2.60)	4.64 (2.91)	< 0.001
Pre-frail	15,745	3.43 (2.66)	3.60 (2.99)	< 0.001	21,543	4.41 (2.66)	4.54 (3.06)	< 0.001
Frail	1864	3.52 (2.75)	3.50 (3.07)	0.79	2948	4.18 (2.65)	4.13 (3.11)	0.45
**Fruit**								
Health	33,212	1.89 (1.43)	1.97 (1.56)	< 0.001	35,067	2.17 (1.29)	2.16 (1.40)	0.004
Pre-frail	14,852	1.84 (1.46)	1.99 (1.63)	< 0.001	20,749	2.11 (2.33)	2.15 (1.46)	0.03
Frail	1728	1.78 (1.49)	1.99 (1.88)	< 0.001	2803	2.04 (1.38)	2.07 (1.61)	0.76
**Grain**								
Health	33,080	5.05 (2.70)	4.96 (2.70)	< 0.001	34,280	4.84 (2.66)	4.61 (2.73)	< 0.001
Pre-frail	14,803	4.94 (2.76)	4.93 (2.80)	0.52	20,136	4.84 (2.73)	4.62 (2.81)	< 0.001
Frail	1723	4.90 (2.85)	5.03 (2.75)	0.01	2698	4.80 (2.90)	4.59 (2.88)	< 0.01
**Lean meats & poultry**								
Health	34,766	6.88 (3.96)	7.31 (3.53)	< 0.001	36,173	6.56 (3.52)	6.91 (3.03)	< 0.001
Pre-frail	15,745	6.80 (4.09)	7.19 (3.93)	< 0.001	21,543	6.51 (3.65)	6.87 (3.21)	< 0.001
Frail	1864	6.70 (3.98)	7.03 (3.79)	0.003	2948	6.46 (3.94)	6.62 (3.24)	0.04
**Alcohol**								
Health	34,466	10.5 (11.2)	9.86 (10.9)	< 0.001	35,707	5.13 (6.08)	4.89 (6.04)	< 0.001
Pre-frail	15,563	10.3 (12.4)	8.74 (11.4)	< 0.001	21,114	4.34 (6.25)	3.81 (5.95)	< 0.001
Frail	1836	10.3 (14.1)	7.25 (11.8)	< 0.001	2883	3.25 (5.91)	2.43 (5.16)	< 0.001
**Dietary risk scores**								
Health	34,766	4.29 (1.48)	4.18 (1.48)	< 0.001	36,173	3.74 (1.46)	3,76 (1.47)	0.02
Pre-frail	15,745	4.30 (1.51)	4.14 (1.50)	< 0.001	21,543	3.75 (1.50)	3.77 (1.49)	0.04
Frail	1864	4.29 (1.52)	4.10 (1.53)	< 0.001	2948	3.81 (1.53)	3.92 (1.53)	< 0.01
		**Yes**	**Yes**			**Yes**	**Yes**	
**Dairy**		N (%)	N (%)			N (%)	N (%)	
Health	–	34,019 (66.4)	33,890 (66.6)	0.63	–	35,392 (59.7)	35,411 (59.7)	0.89
Pre-frail	–	15,372 (30.0)	15,218 (29.9)	0.67	–	21,052 (35.5)	21,008 (35.4)	0.80
Frail	–	1822 (3.56)	1802 (3.54)	0.88	–	2863 (4.83)	2882 (4.86)	0.79
**Food diversity**								
Health	–	25,141 (67.8)	25,351 (67.9)	0.71	–	27,346 (60.8)	26,884 (61.2)	0.30
Pre-frail	–	10,748 (29.0)	10,782 (28.9)	0.78	–	15,603 (34.7)	15,132 (34.4)	0.39
Frail	–	1189 (3.21)	1184 (3.17)	0.79	–	1998 (4.45)	1925 (4.38)	0.64

Significant increases in vegetable consumption (*p* < 0.001) were found for males and females who identified as healthy and pre-frail. There were significant increases in fruit consumption for males (*p* < 0.001), and for females who were identified as pre-frail (*p* = 0.03). Significant decreases on grain consumption were found for males and females who identified as healthy (*p* < 0.001), and for female who identified as pre-frail (*p* < 0.001) and frail (*p* < 0.01), however, significant increases were found for males who identified as frail (*p* = 0.01). Significant increases in lean meats and poultry consumption were found for males and females (*p* < 0.05), while significant decreases of alcohol consumption were observed across three FRAIL categories for males and females (*p* < 0.001). No significant differences were found for dairy and food diversity across two waves for males and females. Males had significant decreases in overall dietary risk scores, while females had significant increases in dietary risk scores across two waves.

### The long-term dietary consumption and frailty

The associations between long-term dietary consumption and frailty are shown in Table 3. After adjustment of socio-economic and health behaviour factors, no significant associations were found between vegetable consumption and frailty for males. Females with a long-term inadequate vegetable consumption (RRR = 1.34, 95% CI: 1.19; 1.51), and those with inadequate vegetable consumption at either wave had a higher risk of frailty, compare to females who had a long-term adequate vegetable consumption.

**Table 3:** The association between a long-term food group consumption and frailty, stratified by sex

Long-term food grouping	Males				
	Relative Risk Ratio (RRR)				
	Crude model			Adjusted model^**a**^	
	Healthy	Pre-frail	Frail	Pre-frail	Frail
**Vegetable**					
+, + (*N* = 4018)	1	1	1	1	1
-, − (*N* = 35,302)	1	**0.86 (0.80; 0.92)**	**0.67 (0.57; 0.79)**	0.99 (0.91; 1.07)	0.96 (0.79; 1.16)
+, − (*N* = 5415)	1	0.98 (0.90; 1.07)	0.94 (0.77; 1.15)	1.00 (0.90; 1.10)	1.05 (0.83; 1.32)
-, + (*N* = 7640)	1	0.96 (0.88; 1.04)	0.82 (0.68; 1.00)	1.03 (0.93; 1.13)	1.08 (0.86; 1.35)
**Fruit**					
+, + (*N* = 19,046)	1	1	1	1	1
-, − (*N* = 14,128)	1	1.05 (1.00; 1.10)	**1.31 (1.15; 1.48)**	1.02 (0.97; 1.08)	1.06 (0.92; 1.23)
+, − (*N* = 6526)	1	**1.10 (1.04; 1.18)**	**1.45 (1.24; 1.69)**	1.06 (0.99; 1.14)	**1.22 (1.02; 1.45)**
-, + (*N* = 7395)	1	**1.21 (1.14; 1.29)**	**1.49 (1.28; 1.72)**	**1.15 (1.07; 1.22)**	**1.29 (1.09; 1.53)**
**Grain**					
+, + (*N* = 23,200)	1	1	1	1	1
-, − (*N* = 14,730)	1	1.00 (0.95; 1.04)	0.91 (0.81; 1.02)	**1.13 (1.07; 1.19)**	**1.19 (1.03; 1.37)**
+, − (*N* = 5016)	1	1.01 (0.95; 1.08)	0.86 (0.72; 1.02)	**1.12 (1.04; 1.21)**	1.10 (0.90; 1.36)
-, + (*N* = 4419)	1	**1.23 (1.15; 1.32)**	**1.48 (1.26; 1.73)**	**1.32 (1.22; 1.43)**	**1.74 (1.44; 2.09)**
**Lean meats & poultry**					
+, + (*N* = 19,663)	1	1	1	1	1
-, − (*N* = 13,944)	1	**1.08 (1.03; 1.14)**	**1.16 (1.02; 1.30)**	1.05 (1.00; 1.11)	1.00 (0.87; 1.15)
+, − (*N* = 8757)	1	**1.10 (1.04; 1.16)**	**1.38 (1.21; 1.58)**	1.04 (0.97; 1.10)	1.11 (0.95; 1.30)
-, + (*N* = 10,011)	1	**1.08 (1.03; 1.14)**	**1.28 (1.12; 1.46)**	**1.07 (1.01; 1.13)**	1.12 (0.96; 1.30)
**Dairy**					
+, + (*N* = 49,950)	1	1	1	1	1
-, − (*N* = 202)	1	1.18 (0.87; 1.59)	1.65 (0.89; 3.06)	1.16 (0.83; 1.63)	1.88 (0.97; 3.65)
+, − (*N* = 1263)	1	**1.37 (1.22; 1.54)**	1.28 (0.96; 1.70)	**1.23 (1.07; 1.41)**	0.84 (0.59; 1.18)
-, + (*N* = 960)	1	1.10 (0.96; 1.26)	0.94 (0.65; 1.35)	1.05 (0.89; 1.22)	0.89 (0.59; 1.35)
**Food diversity**					
+, + (*N* = 30,718)	1	1	1	1	1
-, − (*N* = 4821)	1	**1.20 (1.12; 1.28)**	**1.49 (1.27; 1.74)**	**1.22 (1.13; 1.31)**	**1.50 (1.25; 1.80)**
+, − (*N* = 4127)	1	**1.21 (1.13; 1.30)**	**1.38 (1.16; 1.64)**	**1.24 (1.14; 1.34)**	**1.27 (1.04; 1.56)**
-, + (*N* = 4375)	1	**1.20 (1.12; 1.28)**	**1.42 (1.20; 1.68)**	**1.21 (1.12; 1.31)**	**1.32 (1.08; 1.61)**
**Alcohol consumption**					
+, + (*N* = 8560)	1	1	1	1	1
-, − (*N* = 37,426)	1	**0.68 (0.64; 0.71)**	**0.40 (0.36; 0.45)**	**0.73 (0.69; 0.77)**	**0.49 (0.43; 0.55)**
+, − (*N* = 1672)	1	**0.85 (0.76; 0.95)**	**0.58 (0.44; 0.76)**	0.89 (0.79; 1.02)	**0.64 (0.46; 0.89)**
-, + (*N* = 3243)	1	**1.37 (1.26; 1.49)**	**1.58 (1.35; 1.86)**	**1.28 (1.17; 1.41)**	**1.40 (1.16; 1.69)**
**Females**					
**Vegetable**					
+, + (*N* = 15,274)	1	1	1	1	1
-, − (*N* = 24,738)	1	1.04 (0.99; 1.08)	**1.44 (1.30; 1.59)**	1.03 (0.98; 1.08)	**1.34 (1.19; 1.51)**
+, − (*N* = 9780)	1	**1.08 (1.02; 1.14)**	**1.50 (1.33; 1.69)**	1.02 (0.96; 1.09)	**1.27 (1.10; 1.47)**
-, + (*N* = 10,872)	1	**1.01 (0.95; 1.06)**	**1.14 (1.00; 1.29)**	1.01 (0.96; 1.07)	1.10 (0.95; 1.28)
**Fruit**					
+, + (*N* = 32,170)	1	1	1	1	1
-, − (*N* = 10,167)	1	**1.15 (1.09; 1.20)**	**1.47 (1.33; 1.63)**	**1.10 (1.04; 1.16)**	**1.29 (1.14; 1.46)**
+, − (*N* = 7490)	1	**1.11 (1.05; 1.17)**	**1.60 (1.43; 1.79)**	**1.10 (1.04; 1.17)**	**1.49 (1.30; 1.70)**
-, + (*N* = 6627)	1	**1.16 (1.10; 1.23)**	**1.55 (1.38; 1.75)**	**1.16 (1.09; 1.23)**	**1.60 (1.39; 1.84)**
**Grain**					
+, + (*N* = 22,727)	1	1	1	1	1
-, − (*N* = 19,095)	1	**0.93 (0.90; 0.97)**	**0.88 (0.80; 0.96)**	**1.10 (1.05; 1.15)**	**1.30 (1.16; 1.46)**
+, − (*N* = 7384)	1	1.00 (0.95; 1.06)	1.00 (0.88; 1.13)	**1.15 (1.08; 1.22)**	**1.35 (1.17; 1.57)**
-, + (*N* = 5352)	1	1.03 (0.97; 1.10)	1.05 (0.91; 1.21)	**1.13 (1.05; 1.22)**	**1.35 (1.15; 1.59)**
**Lean meats & poultry**					
+, + (*N* = 22,408)	1	1	1	1	1
-, − (*N* = 17,020)	1	**1.08 (1.03; 1.12)**	**1.22 (1.11; 1.34)**	1.03 (0.98; 1.08)	1.10 (0.98; 1.23)
+, − (*N* = 10,219)	1	**1.16 (1.10; 1.22)**	**1.39 (1.25; 1.55)**	**1.09 (1.03; 1.26)**	**1.22 (1.07; 1.39)**
-, + (*N* = 11,017)	1	**1.11 (1.06; 1.17)**	**1.19 (1.07; 1.33)**	**1.08 (1.02; 1.14)**	1.11 (0.97; 1.26)
**Dairy**					
+, + (*N* = 58,143)	1	1	1	1	1
-, − (*N* = 199)	1	1.22 (0.91; 1.63)	1.25 (0.67; 2.32)	1.19 (0.85; 1.68)	1.53 (0.74; 3.15)
+, − (*N* = 1164)	1	**1.18 (1.04; 1.33)**	1.04 (0.79; 1.38)	0.98 (0.85; 1.13)	**0.60 (0.42; 0.85)**
-, + (*N* = 1158)	1	1.03 (0.91; 1.17)	**1.36 (1.07; 1.74)**	1.05 (0.91; 1.21)	**1.39 (1.04; 1.85)**
**Food diversity**					
+, + (*N* = 36,991)	1	1	1	1	1
-, − (*N* = 5380)	1	**1.15 (1.08; 1.22)**	**1.33 (1.17; 1.52)**	**1.19 (1.11; 1.27)**	**1.42 (1.21; 1.66)**
+, − (*N* = 5434)	1	**1.21 (1.14; 1.29)**	**1.57 (1.38; 1.78)**	**1.27 (1.19; 1.36)**	**1.67 (1.44; 1.94)**
-, + (*N* = 4425)	1	**1.18 (1.10; 1.26)**	**1.53 (1.33; 1.76)**	**1.20 (1.12; 1.29)**	**1.56 (1.33; 1.84)**
**Alcohol consumption**					
+, + (*N* = 18,935)	1	1	1	1	1
-, − (N = 32,160)	1	**0.56 (0.54; 0.58)**	**0.26 (0.24; 0.28)**	**0.63 (0.60; 0.66)**	**0.36 (0.32; 0.40)**
+, − (*N* = 2686)	1	**0.74 (0.68; 0.80)**	**0.51 (0.42; 0.61)**	**0.78 (0.71; 0.86)**	**0.59 (0.47; 0.74)**
-, + (*N* = 4417)	1	0.95 (0.89; 1.12)	0.97 (0.85; 1.10)	0.97 (0.90; 1.05)	1.05 (0.90; 1.22)

^a^after adjustment of socioeconomic factors and health behaviour factors

Compared with males and females who had a long-term fruit consumption, males and females with inadequate fruit consumption at both waves or had inadequate fruit consumption at either wave had a higher risk of frailty.

Compared with males and females with a long-term high grain consumption, males who had a long-term low grain consumption (RRR = 1.19, 95% CI: 1.03; 1.37) and those who had a low grain consumption at baseline but had high grains consumption at follow-up (RRR = 1.74, 95% CI: 1.44; 2.09) had a higher risk of frailty. Females who had a long-term low grains consumption (RRR = 1.30, 95% CI: 1.16; 1.46), and those who had a low grains consumption at either wave (RRR = 1.35, 95% CI: 1.17; 1.57; RRR = 1.35, 95% CI: 1.15; 1.59) had a higher risk of frailty.

Compared with males and females with a long-term high lean meats and poultry consumption, males who had a low lean meats and poultry consumption at baseline but had high lean meats and poultry consumption at follow-up had a higher risk of pre-frailty (RRR = 1.07, 95% CI:1.01; 1.13) Females who had a high lean meats and poultry consumption at baseline but had low lean meats and poultry consumption at follow-up (RRR = 1.22, 95% CI:1.07; 1.39) had a higher risk of frailty.

Compared with males and females with long-term dairy consumption, males who had dairy consumption at baseline but no dairy consumption at follow-up (RRR = 1.23, 95% CI: 1.07; 1.41) had a higher risk of pre-frailty. Females who had dairy consumption at baseline but no dairy consumption at follow-up had a lower risk of frailty (RRR = 0.60, 95% CI: 0.42; 0.85), while those had no dairy consumption at baseline but had dairy consumption at follow-up had a higher risk of frailty (RRR = 1.39, 95% CI:1.04; 1.85).

Males and females who didn’t consume a long-term variety of foods, or only had a variety of food at one wave had a higher risk of frailty than those with a long-term variety of food consumption.

Compared with males and females without alcohol consumption, those with a long-term alcohol consumption (males: RRR = 0.49, 95% CI: 0.43; 0.55; females: RRR = 0.36; 95% CI: 0.32; 0.40) and those with no alcohol consumption at baseline but had alcohol consumption at follow-up had a lower risk of frailty (males: RRR = 0.64, 95% CI: 0.46; 0.89; females: RRR = 0.59, 95% CI: 0.47; 0.74). However, males with alcohol consumption at baseline but has no alcohol consumption has a higher risk of frailty (RRR = 1.40, 95% CI: 1.16; 1.69).

The association between a long-term each food group consumption and each domain of the FRAIL scale, stratified by sex are shown in Table S3a & Table S3b. Overall, the significant associations between food groups and all five domains of FRAIL were observed for males and females, however the associations differed by sex.

The association between overall dietary risk scores and frailty are shown in Table 4. Females with a long-term unhealthy diet at both waves (RRR = 1.32, 95% CI: 1.18; 1.49), had unhealthy diet at either wave (RRR = 1.28, 95% CI: 1.12; 1.47; RRR = 1.19, 95% CI: 1.04; 1.37) had a higher risk of frail than those had a long-term healthy diet. No association were found between total dietary risk and frailty for males.

**Table 4:** The association between overall dietary risk scores and frailty, stratified by sex

Long-term overall dietary grouping	Relative Risk Ratio (RRR)				
	Crude model			Adjusted model^**a**^	
	Healthy	Pre-frail	Frail	Pre-frail	Frail
**Males**					
+, + (*N* = 8343)	1	1	1	1	1
-, − (*N* = 27,675)	1	0.96 (0.91; 1.01)	0.89 (0.78; 1.02)	1.02 (0.96; 1.08)	0.93 (0.79; 1.08)
+, − (*N* = 7327)	1	1.01 (0.95; 1.09)	1.00 (0.84; 1.18)	1.02 (0.95; 1.11)	0.92 (0.76; 1.12)
-, + (*N* = 9030)	1	1.04 (0.98; 1.11)	1.11 (0.95; 1.29)	1.05 (0.98; 1.13)	1.11 (0.93; 1.33)
**Females**					
+, + (*N* = 16,366)	1	1	1	1	1
-, − (*N* = 23,233)	1	1.03 (0.99; 1.08)	**1.24 (1.12; 1.37)**	**1.06 (1.01; 1.11)**	**1.32 (1.18; 1.49)**
+, − (*N* = 10,681)	1	**1.06 (1.01; 1.12)**	**1.31 (1.17; 1.47)**	1.04 (0.99; 1.11)	**1.28 (1.12; 1.47)**
-, + (*N* = 10,384)	1	**1.05 (1.00; 1.11)**	**1.18 (1.05; 1.33)**	1.04 (0.98; 1.10)	**1.19 (1.04; 1.37)**

^a^after adjustment of socioeconomic factors and health behaviour factors

## Discussion

Our results indicate that the prevalence of frailty was higher in females than in males. People aged 80 and over and people living in rural areas have been oversampled, and the sample also skews to higher socioeconomic status. As age increases, males had better dietary behaviour, while females had worse dietary behaviour, which affected their frailty status. Males and females with a long-term consumption of adequate fruits, high grains or had a variety of foods were associated with a lower risk of frailty. Females with a long-term consumption of adequate vegetables or had high lean meats and poultry were associated with a lower risk of frailty. In addition, females with a long-term overall healthy diet were associated with a lower risk of frailty. No association were found between overall dietary risk and frailty for males.

Given it has a well-described clinical phenomenon that females live longer than males yet tend to experience greater levels of co-morbidity and disability, these results highlight the importance of sex related frailty. Our results show that the prevalence of frailty was higher in females than in males, which is consistent with previous studies [19, 20]. For example, a study results from four pooled Australian cohort studies that including 8804 Australian adults aged 65 years and over [22], and a meta-analysis of data from five studies that including 37,426 participants show that females have higher prevalence of frailty than males [23].

Many studies have shown that males and females have different dietary habits or dietary pattern [24]. For example, the results from Foodborne Diseases Active Surveillance Network Population Survey shows that a higher proportion of men reported eating protein than women, whereas a higher proportion of women consumed fruits and vegetables [25]. These dietary habits may further impact on disease status. Our results support this point, highlighting that with age increases, males had significant decreases in dietary risk scores while females had significant increases in dietary risk scores, in particular those who identified as frail. However, reasons to explain this are not clear. It might be due to that females experienced poor health and living with disease than males as they age [26], which may position them at poor nutrition risk. In addition, we found females who with a long-term lower dietary risk scores had a lower risk of frailty, but there was no association for males. It implies that there is a potential of a stronger link between dietary consumption and frailty in females than males, suggesting sex-specific dietary advice need to be further developed in preventing the frailty.

Although studies have proposed consuming fruit and vegetable (FV) may protect against frailty, the overall evidence is scarce with the study settings and findings heterogeneous [27]. A study including three independent cohorts of community dwelling older adults showed that three portions of fruit per day and two portions of vegetables per day had the strongest associated with the lowest short-term risk of frailty [28]. Studies have proposed four potential mechanisms of the benefit of FV in relation to frailty, including 1) antioxidants in FV play a role in slowing frailty development, 2) phytochemicals contained in FV have strong anti-inflammatory properties that heighten inflammatory stares in older people, 3) FV are rich in nutrients, such as dietary fibre, were strongly linked to a lower incidence of certain diseases (e.g., cardiovascular disease), which is one of the indicators in many frailty measurements, and 4) FV consumption has been associated with stimulating the immune system which is strongly associated with showing frailty development [28]. Some studies assessed the association between specific dietary patterns and frailty [29, 30], with FV consumption considered as a sub-analysis, showing that higher FV consumption is associated with a lower incident frailty risk. The sex-specific analysis on the FV and frailty were scarce in the existing literature. Our study showed that the inverse associations between fruits and frailty were observed for both males and females, while the inverse associations between vegetables and frailty were only found for females.

In general, we found dietary behaviours vary as age increases. Many studies have shown that people are likely to make different food choices as they get older [31]. This may be mainly due to the physiological changes that associated with age, such as slower gastric emptying, decreased basal metabolic rate, altered taste and smell. Some other factors have been also noted, such as changes on socioeconomic status and diet-related attitudes and beliefs [31]. These changes may increase the risk of diet-related illness.

With age increases, females had a significant reduction in grains consumption (including both whole grain and refined grains). Our results highlighted the benefit of long-term high grains consumption in relation to frailty for males and females. Given the benefit of a long-term grains consumption and frailty, our results suggested that females need to be encouraged to consume more grains in the prevention of frailty. Grains or cereals consumption, often considered as one component in the specific dietary pattern, has been explored in relation to frailty in the existing literature. A recent system review which includes a total of 13 cohort or cross-sectional studies examined the association between dietary patterns and risk of frailty showed that a diet high in fruit, vegetables and whole grains was associated with reduced risk of frailty [32]. However, the association between grain consumption in different diseases may differ by age groups [33], and different types of grains may play different roles in disease prevention for older people [18]. Therefore, dietary advice on specific type of grain consumption by different age groups, for different diseases, needs to be further investigated.

Although dietary protein has always been encouraged to prevent frailty, we found that as age increases, males and females have a significant increase in their lean meats and poultry consumption. Moreover, the benefit of a high lean meats and poultry consumption in relation to frailty was only observed for females. A few studies indicate a protective role of protein supplementation against frailty syndrome, very little evidence regarding the effect of protein supplementation on frailty [1]. A daily 30 g protein supplement has been suggested to prevent frailty. However, studies have highlighted specific individual characteristics should be considered before prescribing supplements because excess protein can be harmful [1]. Our results suggest that personalized dietary advice, with the person understanding their dietary protein consumption habit, is needed for health professionals in order to provide accurate dietary advice to prevent frailty.

Our results support the previous study that a balanced diet, including consuming a variety of food, is a reasonable approach to prevent frailty. Few studies have indicated that a balance diet could be beneficial in avoiding frailty. For example, adherence to a Mediterranean dietary pattern has been associated with lower odds of frailty [34]. A systematic review including 19 studies, encompassing a sample of 22,270 older adults suggested the importance of both quantitative (energy intake) and qualitative (nutrient quality) factors of nutrition in the development of frailty.. However, most of these studies were of cross-sectional design [3]. We suggest that more longitudinal studies of understanding the potential role of nutrition in prevention, postponement, or even reversion of frailty syndrome are required [3].

Our results also indicate that males and females had a significant decrease on alcohol consumption as they age. In terms of the relationship between alcohol consumption and frailty, there were inconsistent results across the literature. Some studies indicated the benefits of moderate and harms of no alcohol consumption associated with a higher risk of frailty [35–37], which is in line with our study results. However, a study which followed up the participants across their middle and older ages for 30 years highlighted the relationship between alcohol consumption and frailty transforms during the life course. High alcohol consumption in midlife predicts frailty, whereas the association is reversed in old age [38]. It is possible that alcohol consumption at early age can cause health problems at older age.. Older people with no alcohol consumption may have chosen abstinence due to illness and are therefore susceptible for frailty. Given we only can identify frailty at one time point from our data, further studies using more survey points of follow-up data are required to understand the relationship between long-term alcohol consumption and frailty among the older Australian population.

The strength of the present study is that it involved a large population sample. Tracking a long-term dietary consumption, along with sex-specific analyses provided significant insights on the dietary advice in prevention of frailty for middle-aged and older people. However, there are some limitations. Principally this includes the use of self-reported data, which may have measurement bias on dietary consumption. Second, the measures of dietary risk behaviours are limited, and we were not able to calculate overall caloric intake. A short dietary questionnaire does not capture all relevant food. For example, yogurt should belong to the ‘dairy’ group, but we were unable to include it in the analysis..Thirdly, the definition of frailty was identified based on self-reported surveys rather than objective measures. The frailty status can only be identified from the follow-up survey that it is not possible to track the changes of frailty status over time. One of the components in the FRAIL scale is loss of weight. We identified ‘loss of weight’ if participants’ weight decreased by 5% or more between baseline (2006–2009) and follow-up survey (2012–2015). Given the time interval between baseline and follow-up survey, it is hardly to know the exact time when participants losing 5% or more weight. In addition, depending on age and baseline weight, this time interval may carry different clinical significance in identifying frailty. Further studies of including more survey points of follow-up data will be conducted.

In conclusion, our study suggests that males and females changed their dietary consumption as they age, and these changes affect its association with frailty, particularly for females. The general dietary advice to everyone in prevention of frailty may not be enough. Sex-specific dietary advice, by considering the changes of dietary consumption in males and females as they age, in prevention of frailty needs to be further developed. In addition, understanding individual long-term dietary habit for initial assessment can facilitate health professionals to provide accurate healthy dietary advice to prevent frailty.

## Supplementary Information

**Additional file 1: Table S1.** Scoring overall dietary scores. **Table S2.** Group classifications of a long-term dietary consumption. **Table S3a.** The association between a long-term food group consumption and each domain of the FRAIL scale among males. **Table S3b.** The association between a long-term dietary group consumption and each domain of the FRAIL scale among females.

## Data Availability

The 45 and Up Study is not publicly available, and it is managed by the Sax Institute. For data access, please contact the 45 and Up Study team at 45andUp.research@saxinstitute.org.au.
