# Supplementary material for: Sex differences in dietary consumption and its association with frailty among middle-aged and older Australians: a 10-year longitudinal survey
Source: BMC Geriatr. 2021 Mar 31;21:217. doi: 10.1186/s12877-021-02165-2 (PMC8011098; doi:10.1186/s12877-021-02165-2)
Supplement: Supplementary file 1 — Additional file 1: Table S1. Scoring overall dietary scores. Table S2. Group classifications of a long-term dietary consumption. Table S3a. The association between a long-term food group consumption and each domain of the FRAIL scale among males. Table S3b. The association between a long-term dietary group consumption and each domain of the FRAIL scale among females. [file 12877_2021_2165_MOESM1_ESM.docx]

**Sex differences in dietary consumption over life course and its association with frailty among middle-aged and older Australians**

**Xiaoyue Xu^1,2,3*^, Sally Inglis^3^, Deborah Parker^3^**

^1^ School of Population Health, University of New South Wales, New South Wales, Australia

^2^ The George Institute for Global Health, Sydney New South Wales, Australia

^3^ Improving Palliative, Aged and Chronic Care through Clinical Research and Translation Research Centre, Faculty of Health, University of Technology Sydney, New South Wales, Australia

*Corresponding author.

**Table S1. Scoring overall dietary scores**

| **Dietary behaviour** | **Male** | | **Female** | |
| --- | --- | --- | --- | --- |
|  | **Age groups** | | **Age groups** | |
|  | **51-70 years** | **70+ years** | **51-70 years** | **70+ years** |
| Vegetable | ≥5.5 serve per day=0  <5.5 serve per day=1 | ≥5 serve per day=0  <5 serve per day=1 | ≥5 serve per day=0  <5 serve per day=1 | ≥5 serve per day=0  <5 serve per day=1 |
| Fruit | ≥2 serve per day=0  <2 serve per day=1 | | | |
| Grain **^‡^** | High frequency=0  Medium frequency=1  Low frequency=2 | | | |
| Lean meats and poultry**^¶^** | High frequency=0  Medium frequency=1  Low frequency=2 | | | |
| Dairy **^†^** | Yes=0  No=1 | | | |
| Food diversity | Consume all five groups=0, Not consume all five groups=1 | | | |
| Alcohol consumption | ≤10 standard drink per week=0, >10 standard drink per week=1 | | | |

**^‡^** Grain consumption was measured by the times of consumption per week.

**^¶^** Include beef, lamb, pork, chicken, turkey, duck, fish and seafood. Lean meats and poultry consumption were measured by the times of consumption per week.

**^†^** include cheese and milk.

**Table S2. Group classifications of a long-term dietary consumption**

| **Food groups** | **N (%)** | **Grouping** |
| --- | --- | --- |
| **Vegetable*** |  |  |
| Had adequate consumption at baseline and follow-up | 19,292 (17.1) | +, + |
| Had inadequate consumption at baseline and follow-up | 60,040 (53.1) | -, - |
| Had adequate consumption at baseline but inadequate consumption at follow-up | 15,195 (13.4) | +, - |
| Had inadequate consumption at baseline but adequate consumption at follow-up | 18,512 (16.4) | -, + |
| **Fruit**** |  |  |
| Had adequate consumption at baseline and follow-up | 51,216 (49.5) | +, + |
| Had inadequate consumption at baseline and follow-up | 24,295 (23.5) | -, - |
| Had adequate consumption at baseline but inadequate consumption at follow-up | 14,016 (13.5) | +, - |
| Had inadequate consumption at baseline but adequate consumption at follow-up | 14,022 (13.5) | -, + |
| **Grain** |  |  |
| Had higher than mean consumption (>5) at baseline and follow-up | 45,927 (45.1) | +, + |
| Had lower than mean consumption (≤5) at baseline and follow-up | 33,825 (33.2) | -, - |
| Had higher than mean consumption (>5) at baseline but lower than mean consumption (≤5) at follow-up | 12,400 (12.2) | +, - |
| Had lower than mean consumption (≤5) at baseline but higher than mean consumption (>5) at follow-up | 9,771 (9.59) | -, + |
| **Lean meats and poultry** |  |  |
| Had higher than mean consumption (≥7) at baseline and follow-up | 42,071 (37.2) | +, + |
| Had lower than mean consumption (<7) at baseline and follow-up | 30,964 (27.4) | -, - |
| Had higher than mean consumption (≥7) at baseline but lower than mean consumption (<7) at follow-up | 18,976 (16.8) | +, - |
| Had lower than mean consumption (<7) at baseline but higher than mean consumption (≥7) at follow-up | 21,028 (18.6) | -, + |
| **Dairy** |  |  |
| Had consumption at baseline and follow-up | 108,093 (95.6) | +, + |
| No consumption at baseline and follow-up | 401 (0.35) | -, - |
| Had consumption at baseline but no consumption at follow-up | 2,427 (2.15) | +, - |
| No consumption at baseline but had consumption at follow-up | 2,118 (1.87) | -, + |
| **Food diversity** |  |  |
| Had all five groups consumption at baseline and follow-up | 67,709 (70.3) | +, + |
| Had no five groups consumption at baseline and follow-up | 10,201 (10.6) | -, - |
| Had five groups consumption at baseline but no five groups consumption at follow-up | 9,561 (9.93) | +, - |
| Had no five groups consumption at baseline but had five groups consumption at follow-up | 8,800 (9.14) | -, + |
| **Alcohol consumption** |  |  |
| No consumption at baseline and follow-up | 27,495 (25.2) | +, + |
| Had consumption at baseline and follow-up | 69,586 (63.8) | -, - |
| No consumption at baseline but had consumption at follow-up | 4,358 (3.99) | +, - |
| Had consumption at baseline but no drinking at follow-up | 7,660 (7.02) | -, + |
| **Overall dietary risk** |  |  |
| Had lower than mean scores (<4) at baseline and follow-up | 24,709 (21.9) | +, + |
| Had higher than mean scores (4-9) at baseline and follow-up | 50,908 (45.0) | -, - |
| Had lower than mean scores (<4) at baseline but higher than mean scores (4-9) at follow-up | 18,008 (15.9) | +, - |
| Had higher than mean scores (4-9) at baseline but lower than mean scores (<4) at follow-up | 19,414 (17.2) | -, + |

* Adequate vegetable consumption was identified as ≥5.5 serves per day for males aged 51-70 years, ≥5 serves per day for males who aged 70 and above; and ≥5 serves per day for females across all age groups.

** Adequate fruit consumption was identified as ≥2 serves per day for males and females across all age groups

**Table S3a. The association between a long-term food group consumption and each domain of the FRAIL scale among males**

| **Food groups** | **Fatigue** | | | |
| --- | --- | --- | --- | --- |
|  | **Long-term food grouping** | | | |
| **Vegetable** | **+,+** | **-,-** | **+,-** | **-,+** |
| Crude model | 1 | 1.05 (0.90; 1.22) | 1.16 (0.96; 1.40) | 1.01 (0.84; 1.21) |
| Adjusted model | 1 | 1.15 (0.97; 1.38) | 1.23 (1.00; 1.52) | 1.11 (0.91; 1.36) |
| **Fruit** |  |  |  |  |
| Crude model | 1 | **1.38 (1.25; 1.53)** | **1.37 (1.20; 1.56)** | **1.30 (1.14; 1.47)** |
| Adjusted model | 1 | **1.17 (1.04; 1.31)** | **1.22 (1.05; 1.41)** | 1.15 (0.99; 1.32) |
| **Grain** |  |  |  |  |
| Crude model | 1 | 1.09 (0.99; 1.20) | 1.17 (1.02; 1.34) | **1.35 (1.17; 1.55)** |
| Adjusted model | 1 | 1.04 (0.93; 1.16) | 1.16 (0.99; 1.36) | **1.31 (1.13; 1.53)** |
| **Lean meats and poultry** |  |  |  |  |
| Crude model | 1 | **1.16 (1.04; 1.28)** | **1.13 (1.00; 1.27)** | **1.13 (1.01; 1.26)** |
| Adjusted model | 1 | 1.11 (0.99; 1.24) | 1.12 (0.98; 1.27) | 1.11 (0.98; 1.26) |
| **Dairy** |  |  |  |  |
| Crude model | 1 | 1.35 (0.75; 2.42) | **1.50 (1.12; 1.99)** | **1.37 (1.06; 1.78)** |
| Adjusted model | 1 | 1.23 (0.64; 2.37) | 1.30 (0.93; 1.81) | **1.40 (1.05; 1.86)** |
| **Food diversity** |  |  |  |  |
| Crude model | 1 | **1.54 (1.35; 1.75)** | **1.57 (1.37; 1.81)** | **1.45 (1.26; 1.67)** |
| Adjusted model | 1 | **1.36 (1.17; 1.58)** | **1.45 (1.24; 1.69)** | **1.24 (1.06; 1.46)** |
| **Alcohol consumption** |  |  |  |  |
| Crude model | 1 | **0.49 (0.45; 0.54)** | 0.92 (0.75; 1.13) | 1.16 (1.00; 1.34) |
| Adjusted model | 1 | **0.56 (0.50; 0.62)** | 1.01 (0.81; 1.27) | 1.11 (0.94; 1.31) |
|  | **Resistance** | | | |
| **Vegetable** |  |  |  |  |
| Crude model | 1 | **0.72 (0.66; 0.78)** | 0.95 (0.85; 1.05) | **0.83 (0.75; 0.92)** |
| Adjusted model | 1 | 0.95 (0.85; 1.05) | 1.01 (0.89; 1.14) | 0.98 (0.87; 1.11) |
| **Fruit** |  |  |  |  |
| Crude model | 1 | **1.20 (1.12; 1.27)** | **1.24 (1.14; 1.34)** | **1.29 (1.19; 1.39)** |
| Adjusted model | 1 | **1.12 (1.04; 1.21)** | **1.16 (1.06; 1.28)** | **1.20 (1.10; 1.31)** |
| **Grain** |  |  |  |  |
| Crude model | 1 | 1.02 (0.96; 1.08) | 0.99 (0.91; 1.08) | **1.13 (1.03; 1.23)** |
| Adjusted model | 1 | **1.32 (1.22; 1.42)** | **1.19 (1.07; 1.33)** | **1.27 (1.14; 1.41)** |
| **Lean meats and poultry** |  |  |  |  |
| Crude model | 1 | **1.08 (1.01; 1.14)** | **1.22 (1.14; 1.31)** | **1.10 (1.03; 1.18)** |
| Adjusted model | 1 | 0.99 (0.92; 1.06) | 1.08 (0.99; 1.17) | 1.01 (0.93; 1.09) |
| **Dairy** |  |  |  |  |
| Crude model | 1 | **1.55 (1.09; 2.20)** | **1.74 (1.50; 2.02)** | 1.08 (0.90; 1.29) |
| Adjusted model | 1 | **1.68 (1.12; 2.53)** | **1.39 (1.15; 1.67)** | 0.95 (0.77; 1.18) |
| **Food diversity** |  |  |  |  |
| Crude model | 1 | **1.37 (1.26; 1.48)** | **1.28 (1.17; 1.40)** | **1.20 (1.10; 1.31)** |
| Adjusted model | 1 | **1.44 (1.30; 1.58)** | **1.24 (1.11; 1.38)** | **1.21 (1.09; 1.35)** |
| **Alcohol consumption** |  |  |  |  |
| Crude model | 1 | **0.54 (0.51; 0.58)** | **0.78 (0.68; 0.90)** | **1.28 (1.16; 1.41)** |
| Adjusted model | 1 | **0.63 (0.59; 0.68)** | 0.89 (0.75; 1.05) | **1.13 (1.01; 1.27)** |
|  | **Ambulation** | | | |
| **Vegetable** |  |  |  |  |
| Crude model | 1 | **0.74 (0.66; 0.82)** | 1.00 (0.88; 1.14) | **0.87 (0.77; 0.98)** |
| Adjusted model | 1 | 0.99 (0.87; 1.13) | 1.08 (0.92; 1.26) | 1.07 (0.92; 1.25) |
| **Fruit** |  |  |  |  |
| Crude model | 1 | **1.20 (1.11; 1.30)** | **1.27 (1.15; 1.41)** | **1.29 (1.17; 1.41)** |
| Adjusted model | 1 | 1.08 (0.98; 1.18) | **1.17 (1.04; 1.32)** | **1.18 (1.06; 1.32)** |
| **Grain** |  |  |  |  |
| Crude model | 1 | 1.02 (0.95; 1.10) | 0.98 (0.88; 1.09) | 1.26 (1.13; 1.40) |
| Adjusted model | 1 | **1.38 (1.26; 1.51)** | **1.23 (1.08; 1.41)** | **1.46 (1.28; 1.66)** |
| **Lean meats and poultry** |  |  |  |  |
| Crude model | 1 | **1.18 (1.09; 1.27)** | **1.32 (1.21; 1.44)** | **1.20 (1.11; 1.31)** |
| Adjusted model | 1 | 1.09 (0.99; 1.19) | **1.12 (1.01; 1.25)** | 1.10 (0.99; 1.22) |
| **Dairy** |  |  |  |  |
| Crude model | 1 | **1.85 (1.24; 2.75)** | **1.81 (1.51; 2.16)** | 1.03 (0.82; 1.29) |
| Adjusted model | 1 | **2.02 (1.27; 3.22)** | **1.45 (1.17; 1.81)** | 0.92 (0.70; 1.21) |
| **Food diversity** |  |  |  |  |
| Crude model | 1 | **1.39 (1.25; 1.54)** | **1.35 (1.21; 1.51)** | **1.30 (1.17; 1.45)** |
| Adjusted model | 1 | **1.45 (1.28; 1.63)** | **1.28 (1.12; 1.46)** | **1.30 (1.14; 1.48)** |
| **Alcohol consumption** |  |  |  |  |
| Crude model | 1 | **0.53 (0.49; 0.57)** | **0.75 (0.63; 0.89)** | **1.38 (1.23; 1.55)** |
| Adjusted model | 1 | **0.65 (0.60; 0.71)** | **0.79 (0.64; 0.98)** | **1.24 (1.08; 1.43)** |
|  | **Illness** | | | |
| **Vegetable** |  |  |  |  |
| Crude model | 1 | **0.65 (0.46; 0.92)** | 0.92 (0.60; 1.41) | 0.90 (0.60; 1.34) |
| Adjusted model | 1 | 0.72 (0.50; 1.05) | 0.75 (0.46; 1.22) | 0.92 (0.60; 1.43) |
| **Fruit** |  |  |  |  |
| Crude model | 1 | 0.79 (0.60; 1.04) | 1.20 (0.88; 1.64) | 0.95 (0.68; 1.31) |
| Adjusted model | 1 | **0.60 (0.44; 0.81)** | 0.93 (0.66; 1.31) | 0.69 (0.48; 1.00) |
| **Grain** |  |  |  |  |
| Crude model | 1 | 0.98 (0.76; 1.27) | 0.97 (0.67; 1.42) | 1.27 (0.89; 1.82) |
| Adjusted model | 1 | 0.99 (0.74; 1.33) | 1.04 (0.69; 1.58) | 1.26 (0.85; 1.85) |
| **Lean meats and poultry** |  |  |  |  |
| Crude model | 1 | 0.85 (0.65; 1.12) | 1.23 (0.93; 1.63) | 1.05 (0.79; 1.39) |
| Adjusted model | 1 | 0.77 (0.57; 1.04) | 1.05 (0.77; 1.44) | 0.89 (0.65; 1.22) |
| **Dairy** |  |  |  |  |
| Crude model | 1 | - | 0.79 (0.37; 1.66) | 1.33 (0.69; 1.59) |
| Adjusted model | 1 | - | 0.33 (0.11; 1.03) | 1.14 (0.53; 2.43) |
| **Food diversity** |  |  |  |  |
| Crude model | 1 | 1.22 (0.86; 1.73) | 1.35 (0.94; 1.93) | 1.12 (0.77; 1.64) |
| Adjusted model | 1 | 1.04 (0.71; 1.54) | 1.12 (0.74; 1.67) | 0.96 (0.63; 1.45) |
| **Alcohol consumption** |  |  |  |  |
| Crude model | 1 | **0.42 (0.33; 0.53)** | 0.89 (0.54; 1.48) | 1.08 (0.75; 1.55) |
| Adjusted model | 1 | **0.48 (0.37; 0.62)** | 0.92 (0.53; 1.61) | 1.01 (0.68; 1.48) |
|  | **Loss of weight** | | | |
| **Vegetable** |  |  |  |  |
| Crude model | 1 | **0.92 (0.85; 1.00)** | 1.00 (0.90; 1.11) | 1.04 (0.94; 1.14) |
| Adjusted model | 1 | 1.02 (0.93; 1.12) | 1.00 (0.90; 1.13) | 1.08 (0.97; 1.20) |
| **Fruit** |  |  |  |  |
| Crude model | 1 | 0.95 (0.90; 1.01) | 1.01 (0.94; 1.09) | **1.18 (1.10; 1.25)** |
| Adjusted model | 1 | 0.95 (0.90; 1.02) | 0.98 (0.91; 1.07) | **1.14 (1.06; 1.23)** |
| **Grain** |  |  |  |  |
| Crude model | 1 | **0.89 (0.84; 0.94)** | **0.90 (0.84; 0.98)** | **1.27 (1.18; 1.38)** |
| Adjusted model | 1 | 0.99 (0.93; 1.06) | 1.00 (0.92; 1.10) | **1.36 (1.25; 1.48)** |
| **Lean meats and poultry** |  |  |  |  |
| Crude model | 1 | **1.10 (1.03; 1.16)** | **1.09 (1.02; 1.16)** | 1.06 (1.00; 1.13) |
| Adjusted model | 1 | **1.07 (1.01; 1.14)** | 1.03 (0.96; 1.11) | 1.05 (0.98; 1.12) |
| **Dairy** |  |  |  |  |
| Crude model | 1 | 1.13 (0.80; 1.60) | **1.46 (1.28; 1.66)** | 1.01 (0.85; 1.19) |
| Adjusted model | 1 | 1.13 (0.77; 1.65) | **1.31 (1.13; 1.52)** | 1.05 (0.88; 1.26) |
| **Food diversity** |  |  |  |  |
| Crude model | 1 | 1.00 (0.92; 1.08) | 1.05 (0.97; 1.14) | **1.13 (1.04; 1.22)** |
| Adjusted model | 1 | 1.04 (0.95; 1.13) | **1.10 (1.01; 1.21)** | 1.16 (1.07; 1.27) |
| **Alcohol consumption** |  |  |  |  |
| Crude model | 1 | **0.81 (0.76; 0.86)** | **0.79 (0.69; 0.91)** | **1.47 (1.34; 1.62)** |
| Adjusted model | 1 | **0.82 (0.77; 0.87)** | **0.81 (0.69; 0.94)** | **1.39 (1.25; 1.54)** |

**Table S3b. The association between a long-term dietary group consumption and each domain of the FRAIL scale among females**

| **Food groups** | **Fatigue** | | | |
| --- | --- | --- | --- | --- |
|  | **Long-term food grouping** | | | |
| **Vegetable** | **+,+** | **-,-** | **+,-** | **-,+** |
| Crude model | 1 | **1.45 (1.33; 1.58)** | **1.34 (1.21; 1.49)** | 1.10 (0.99; 1.22) |
| Adjusted model | 1 | **1.34 (1.22; 1.48)** | **1.25 (1.11; 1.41)** | 1.06 (0.94; 1.20) |
| **Fruit** |  |  |  |  |
| Crude model | 1 | **1.61 (1.47; 1.75)** | **1.45 (1.31; 1.60)** | **1.44 (1.29; 1.59)** |
| Adjusted model | 1 | **1.37 (1.24; 1.51)** | **1.34 (1.20; 1.50)** | **1.30 (1.15; 1.46)** |
| **Grain** |  |  |  |  |
| Crude model | 1 | **1.28 (1.18; 1.38)** | **1.26 (1.13; 1.40)** | 1.11 (0.98; 1.26) |
| Adjusted model | 1 | **1.24 (1.13; 1.36)** | **1.22 (1.08; 1.38)** | 1.08 (0.94; 1.24) |
| **Lean meats and poultry** |  |  |  |  |
| Crude model | 1 | **1.33 (1.23; 1.44)** | **1.28 (1.17; 1.41)** | **1.11 (1.01; 1.22)** |
| Adjusted model | 1 | **1.26 (1.15; 1.38)** | **1.21 (1.09; 1.34)** | 1.07 (0.96; 1.19) |
| **Dairy** |  |  |  |  |
| Crude model | 1 | **1.69 (1.05; 2.73)** | **1.39 (1.08; 1.78)** | **1.34 (1.08; 1.65)** |
| Adjusted model | 1 | **1.81 (1.07; 3.09)** | 1.19 (0.89; 1.60) | 1.23 (0.97; 1.56) |
| **Food diversity** |  |  |  |  |
| Crude model | 1 | **1.57 (1.41; 1.75)** | **1.50 (1.35; 1.67)** | **1.47 (1.31; 1.65)** |
| Adjusted model | 1 | **1.40 (1.24; 1.58)** | **1.42 (1.26; 1.60)** | **1.34 (1.18; 1.53)** |
| **Alcohol consumption** |  |  |  |  |
| Crude model | 1 | **0.48 (0.44; 0.51)** | **0.83 (0.71; 0.96)** | 0.95 (0.84; 1.06) |
| Adjusted model | 1 | **0.53 (0.49; 0.57)** | 0.85 (0.72; 1.00) | 0.94 (0.82; 1.07) |
|  | **Resistance** | | | |
| **Vegetable** |  |  |  |  |
| Crude model | 1 | **1.19 (1.13; 1.25)** | **1.25 (1.18; 1.33)** | **1.05 (0.99; 1.12)** |
| Adjusted model | 1 | **1.21 (1.14; 1.29)** | **1.15 (1.07; 1.24)** | 1.07 (0.99; 1.15) |
| **Fruit** |  |  |  |  |
| Crude model | 1 | **1.34 (1.27; 1.42)** | **1.30 (1.22; 1.38)** | **1.24 (1.16; 1.32)** |
| Adjusted model | 1 | **1.28 (1.20; 1.37)** | **1.25 (1.16; 1.34)** | **1.26 (1.16; 1.34)** |
| **Grain** |  |  |  |  |
| Crude model | 1 | **0.90 (0.86; 0.94)** | 0.97 (0.91; 1.04) | 1.02 (0.95; 1.09) |
| Adjusted model | 1 | **1.22 (1.15; 1.29)** | **1.22 (1.13; 1.32)** | **1.17 (1.07; 1.28)** |
| **Lean meats and poultry** |  |  |  |  |
| Crude model | 1 | 1.05 (0.99; 1.10) | **1.22 (1.16; 1.30)** | **1.09 (1.03; 1.16)** |
| Adjusted model | 1 | 0.98 (0.93; 1.04) | **1.13 (1.05; 1.21)** | 1.06 (0.99; 1.13) |
| **Dairy** |  |  |  |  |
| Crude model | 1 | 1.08 (0.77; 1.53) | **1.20 (1.04; 1.39)** | 1.14 (0.99; 1.31) |
| Adjusted model | 1 | 1.13 (0.73; 1.75) | 0.89 (0.74; 1.07) | 1.15 (0.97; 1.36) |
| **Food diversity** |  |  |  |  |
| Crude model | 1 | **1.14 (1.07; 1.23)** | **1.19 (1.11; 1.27)** | **1.27 (1.17; 1.36)** |
| Adjusted model | 1 | **1.24 (1.14; 1.35)** | **1.25 (1.15; 1.35)** | **1.31 (1.20; 1.43)** |
| **Alcohol consumption** |  |  |  |  |
| Crude model | 1 | **0.41 (0.40; 0.43)** | **0.66 (0.60; 0.73)** | **0.86 (0.80; 0.93)** |
| Adjusted model | 1 | **0.52 (0.50; 0.55)** | **0.77 (0.68; 0.86)** | **0.90 (0.83; 0.99)** |
|  | **Ambulation** | | | |
| **Vegetable** |  |  |  |  |
| Crude model | 1 | **1.25 (1.17; 1.34)** | **1.35 (1.25; 1.46)** | 1.07 (0.99; 1.16) |
| Adjusted model | 1 | **1.21 (1.12; 1.31)** | **1.19 (1.08; 1.31)** | 1.07 (0.98; 1.18) |
| **Fruit** |  |  |  |  |
| Crude model | 1 | **1.34 (1.25; 1.44)** | **1.37 (1.27; 1.47)** | **1.29 (1.19; 1.40)** |
| Adjusted model | 1 | **1.24 (1.14; 1.35)** | **1.27 (1.16; 1.40)** | **1.30 (1.18; 1.43)** |
| **Grain** |  |  |  |  |
| Crude model | 1 | **0.88 (0.83; 0.94)** | 0.98 (0.90; 1.06) | 1.08 (0.99; 1.19) |
| Adjusted model | 1 | **1.31 (1.21; 1.41)** | **1.37 (1.24; 1.51)** | **1.42 (1.27; 1.58)** |
| **Lean meats and poultry** |  |  |  |  |
| Crude model | 1 | **1.17 (1.10; 1.25)** | **1.31 (1.22; 1.41)** | **1.18 (1.10; 1.26)** |
| Adjusted model | 1 | 1.09 (0.99; 1.19) | **1.12 (1.01; 1.25)** | 1.10 (1.00; 1.22) |
| **Dairy** |  |  |  |  |
| Crude model | 1 | 1.18 (0.78; 1.80) | **1.33 (1.12; 1.59)** | **1.27 (1.07; 1.51)** |
| Adjusted model | 1 | 1.19 (0.68; 2.08) | 0.95 (0.76; 1.20) | 1.18 (0.95; 1.46) |
| **Food diversity** |  |  |  |  |
| Crude model | 1 | 1.17 (1.07; 1.27) | 1.31 (1.21; 1.43) | 1.40 (1.27; 1.53) |
| Adjusted model | 1 | 1.24 (1.11; 1.38) | 1.41 (1.27; 1.57) | 1.48 (1.32; 1.65) |
| **Alcohol consumption** |  |  |  |  |
| Crude model | 1 | **0.34 (0.32; 0.36)** | **0.61 (0.54; 0.69)** | **0.84 (0.77; 0.92)** |
| Adjusted model | 1 | **0.45 (0.42; 0.49)** | **0.71 (0.61; 0.83)** | 0.91 (0.82; 1.12) |
|  | **Illness** | | | |
| **Vegetable** |  |  |  |  |
| Crude model | 1 | 1.20 (0.95; 1.52) | 1.19 (0.89; 1.59) | 1.22 (0.92; 1.62) |
| Adjusted model | 1 | 1.08 (0.83; 1.41) | 1.12 (0.81; 1.54) | 1.11 (0.81; 1.52) |
| **Fruit** |  |  |  |  |
| Crude model | 1 | 1.16 (0.89; 1.50) | **1.33 (1.01; 1.76)** | 1.28 (0.95; 1.72) |
| Adjusted model | 1 | 0.93 (0.69; 1.26) | 1.26 (0.93; 1.71) | 1.17 (0.84; 1.62) |
| **Grain** |  |  |  |  |
| Crude model | 1 | 0.97 (0.77; 1.22) | 1.14 (0.85; 1.52) | 1.09 (0.78; 1.52) |
| Adjusted model | 1 | 1.11 (0.85; 1.44) | 1.26 (0.90; 1.75) | 1.10 (0.75; 1.61) |
| **Lean meats and poultry** |  |  |  |  |
| Crude model | 1 | 1.03 (0.81; 1.29) | 1.21 (0.94; 1.57) | 1.19 (0.92; 1.53) |
| Adjusted model | 1 | 0.91 (0.69; 1.18) | 1.12 (0.84; 1.50) | 1.09 (0.82; 1.45) |
| **Dairy** |  |  |  |  |
| Crude model | 1 | 1.95 (0.62; 6.13) | 0.88 (0.44; 1.78) | 1.34 (0.75; 2.38) |
| Adjusted model | 1 | 2.50 (0.79; 7.94) | 0.61 (0.25; 1.47) | 1.20 (0.61; 2.34) |
| **Food diversity** |  |  |  |  |
| Crude model | 1 | **1.41 (1.03; 1.92)** | 1.11 (0.79; 1.55) | **1.57 (1.14; 2.17)** |
| Adjusted model | 1 | **1.42 (1.00; 2.00)** | 1.09 (0.75; 1.59) | **1.44 (1.00; 2.06)** |
| **Alcohol consumption** |  |  |  |  |
| Crude model | 1 | **0.33 (0.27; 0.41)** | **0.60 (0.38; 0.95)** | 0.84 (0.61; 1.16) |
| Adjusted model | 1 | **0.44 (0.35; 0.56)** | 0.58 (0.34; 1.01) | 0.81 (0.56; 1.17) |
|  | **Loss of weight** | | | |
| **Vegetable** |  |  |  |  |
| Crude model | 1 | 0.96 (0.92; 1.01) | 1.03 (0.97; 1.10) | 1.01 (0.95; 1.07) |
| Adjusted model | 1 | 0.95 (0.90; 1.00) | 1.00 (0.93; 1.07) | 1.02 (0.95; 1.09) |
| **Fruit** |  |  |  |  |
| Crude model | 1 | 0.95 (0.90; 1.01) | 1.00 (0.94; 1.07) | **1.10 (1.03; 1.18)** |
| Adjusted model | 1 | **0.93 (0.87; 0.99)** | 1.02 (0.95; 1.09) | **1.12 (1.04; 1.20)** |
| **Grain** |  |  |  |  |
| Crude model | 1 | **0.84 (0.80; 0.89)** | **0.92 (0.86; 0.98)** | 1.01 (0.94; 1.09) |
| Adjusted model | 1 | 0.96 (0.91; 1.02) | 1.01 (0.94; 1.09) | **1.10 (1.02; 1.20)** |
| **Lean meats and poultry** |  |  |  |  |
| Crude model | 1 | **1.07 (1.02; 1.13)** | **1.13 (1.07; 1.20)** | **1.09 (1.03; 1.15)** |
| Adjusted model | 1 | **1.06 (1.00; 1.12)** | **1.09 (1.02; 1.17)** | **1.08 (1.02; 1.15)** |
| **Dairy** |  |  |  |  |
| Crude model | 1 | 1.35 (0.97; 1.87) | **1.40 (1.22; 1.60)** | 0.97 (0.84; 1.12) |
| Adjusted model | 1 | 1.37 (0.94; 2.00) | **1.23 (1.05; 1.44)** | 1.00 (0.84; 1.17) |
| **Food diversity** |  |  |  |  |
| Crude model | 1 | 1.02 (0.95; 1.09) | **1.21 (1.13; 1.30)** | 1.03 (0.95; 1.11) |
| Adjusted model | 1 | 1.05 (0.97; 1.14) | **1.26 (1.17; 1.37)** | 1.05 (0.96; 1.14) |
| **Alcohol consumption** |  |  |  |  |
| Crude model | 1 | **0.74 (0.71; 0.78)** | **0.78 (0.70; 0.86)** | **1.12 (1.04; 1.21)** |
| Adjusted model | 1 | **0.79 (0.75; 0.83)** | **0.82 (0.73; 0.92)** | **1.12 (1.02; 1.22)** |
